# Adsorption Characteristics and Mechanism of Bisphenol A by Magnetic Biochar

**DOI:** 10.3390/ijerph17031075

**Published:** 2020-02-08

**Authors:** Jinpeng Wang, Ming Zhang

**Affiliations:** 1 College of Biological and Chemical Engineering, Anhui Polytechnic University, Wuhu 241000, China; wangjinpengv3@126.com; 2 School of Architecture and Civil Engineering, Anhui Polytechnic University, Wuhu 241000, China

**Keywords:** biochar, magnetic biochar, adsorption, bisphenol A, γ-Fe_2_O_3_

## Abstract

In this paper, biochar (BC) was prepared from discarded grapefruit peel and modified to prepare magnetic biochar (MBC). Physical and chemical properties of BC and MBC were characterized, and the results showed that the type of iron oxide loaded by MBC was γ-Fe_2_O_3_. Compared with BC, MBC has a larger specific surface area and pore volume, with more oxygen-containing functional groups on the surface. BC and MBC were used to adsorb and remove endocrine-disrupting chemical (EDC) bisphenol A (BPA) from simulated wastewater. The results showed that the adsorption kinetics and adsorption isotherm of BPA adsorption by BC and MBC were mainly in accordance with the pseudo-second-order kinetics model and the Langmuir model. This indicates that the adsorption of BPA on BC and MBC is mainly a chemically controlled monolayer adsorption. Adsorption thermodynamics show that BC and MBC adsorption of BPA is a spontaneous exothermic reaction, and lowering the temperature is conducive to the adsorption reaction. The effect of solution pH on the adsorption of BPA by both was significant. The optimum pH for BC and MBC to absorb BPA was 6 and 3, respectively. The concentration of Na^+^ in the range of 0–0.10 mol·L^−1^ can promote the adsorption of BPA to MBC. MBC loaded with γ-Fe_2_O_3_ not only has excellent magnetic separation ability, but can also reach about 80% of the initial adsorption capacity after four cycles of adsorption. By analyzing the adsorption mechanism, it was found that the H-bond and the π–π electron donor–acceptor interaction (EDA) were the main forces for BC and MBC to adsorb BPA.

## 1. Introduction

Bisphenol A (BPA) is an emerging contaminant that is often detected in natural waters at levels of ng·L^−1^ or ug·L^−1^, making it a trace organic contaminant. Bisphenol A in natural water can cause damage to the reproductive systems of humans or animals. Therefore, scholars generally classify bisphenol A as endocrine-disrupting chemicals (EDCs) [1]. Exposure to endocrine disruptors such as bisphenol A can harm the body. It not only interferes with the male or female reproductive system, but also causes other functional disorders, such as abnormal reproductive function, infertility, precocity, and so on. Affecting the secretion of hormones can also lead to thyroid dysfunction and obesity, which are metabolic disorders [2]. The damage and dysfunction are life-long. In past reports, bisphenol A has often been detected in phenolic wastewater [3,4]. As a monomer of many synthetic materials, bisphenol A has been widely used in diverse industries. For example, bisphenol A can be used to synthesize epoxy resins, polycarbonate [5], phenolic resins, polyester fibers, food cans, and paint coatings [6]. The Brazilian government has banned the production and sale of plastic bottles containing bisphenol A since January 2012. Due to its high industrial yield, environmental universality, and toxicological effect, bisphenol A has been listed as a priority pollutant in water treatment in more and more countries and regions [7].

Bisphenol A in the environment can be released into the water body through the polycarbonate plastic’s natural degradation [8]. Bisphenol A in plastic products can hydrolyze and contaminate groundwater through landfill leachate [9]. The half-life of bisphenol A in the environment is around 4.5 d [10], mainly through the biodegradation of bacteria removal [11]. Studies have shown that due to the anaerobic or hypoxic state of water, the degradation of bisphenol A in natural water is slow, or does not even occur [12]. Therefore, the concentration of bisphenol A in surface water fluctuates greatly depending on the region and discharge time [13]. Coagulation, flocculation, precipitation, and other separation methods have been used to remove bisphenol A from water, while traditional biological treatment methods, such as activated sludge, constructed wetlands, and biological filter, have only a limited effect on the removal of bisphenol A [14,15]. Some relatively advanced treatment technologies, such as photocatalysis, fenton oxidation, membrane separation, and ozone oxidation, have a good removal effect on bisphenol A [14,15,16,17,18]. However, the above treatment technology also has shortcomings, such as high capital costs (CAPEX) and high operating costs, which limits its application in developing countries. As a water treatment process, adsorption has the characteristics of a good effect, an easy operation, and low cost for bisphenol A [19,20]. There have been many studies on endocrine-disrupting chemicals (EDCs) of carbon-based materials, such as activated carbon [21,22,23], carbon black, carbon nanotubes [24,25], and biochar [26,27]. Biochar is a low-cost adsorbent that can effectively remove many hydrophilic and hydrophobic organic pollutants [28,29]. At the same time, after pyrolysis or elution, biochar can be used again to absorb pollutants in water; hence, it has a great application value. However, the small size of biochar particles makes it difficult to recover and regenerate biochar after adsorption, which limits its recycling ability [30].

To solve the problem that biochar is difficult to recover after the adsorption of pollutants, some scholars proposed that biochar with magnetic properties can be prepared by chemical methods. While improving the adsorption performance of biochar, saturated adsorption biochar can be quickly recovered from water by a simple external magnetic field [31]. Grapefruit peel is a kind of porous biomass rich in cellulose and pectin [32]. The annual output of pomelo peel is huge, but only a small amount of pomelo peel is reprocessed into medicine or used as chemical raw materials, and most pomelo peel is treated as agricultural waste, resulting in a great waste of resources and environmental pollution [33]. There are few studies on the adsorption of bisphenol A by magnetic biochar, and its adsorption mechanism has not been elucidated. Therefore, in this study, grapefruit peel waste was used as the carbon source of biochar, and the original biochar was prepared. Magnetic biochar was also prepared by chemical precipitation. The effects of dosage, time, initial bisphenol A concentration, temperature, pH, and ionic strength on the adsorption of bisphenol A by biochar and magnetic biochar were investigated. The mechanism of adsorption of bisphenol A by biochar and magnetic biochar was discussed in combination with the characterization of the original biochar and magnetic biochar, so as to enrich the research of the adsorption of bisphenol A by biochar and magnetic biochar.

## 2. Materials and Methods

### 2.1. Chemicals and Materials

The bisphenol A (98% purity) reagent was purchased from the Aladdin pharmaceutical company (Shanghai, China). Its physical and chemical properties are shown in Table 1. FeSO_4_·7H_2_O and FeCl_3_·6H_2_O were analytically pure (AR). The experimental water was ultrapure water (UP). In order to prevent potential microbial degradation of bisphenol A, the bisphenol A solution used in the experiment was prepared before each experiment.

### 2.2. Biochar Preparation and Modification

The washed and dried grapefruit peel was used as biomass material for pyrolysis at 400 °C for 2 h. We took out the pomelo peel biochar after pyrolysis, ground it, and passed it through a 60-mesh sieve. The seal prepared biochar was put in a plastic bottle labeled “BC” for later use. We took 5 g of the prepared pomelo peel biochar, soaked it in 100 mL of FeSO_4_ of 0.125 mol·L^−1^ and 0.25 mol·L^−1^ of FeCl_3_ solution, and mixed it evenly on the magnetic mixer. Then, we gradually added 100 mL of 1 mol·L^−1^ of NaOH solution. After the reaction was complete, we continued stirring for 1 h. Then, we separated it with a magnetic field and washed it with ultra-pure water until the pH of the solution system was close to neutral. After drying and grinding, the seal prepared magnetic biochar was put in a plastic bottle labeled “MBC” for later use.

### 2.3. Characterization of BC and MBC

The surface structure and morphology of biochar were observed by scanning electron microscopy (SEM, MLA650F, Hillsboro, OR, USA). The contents of C, O, and Fe were determined by X-ray photoelectron spectroscopy (XPS, ESCALAB250). The surface area of biochar was determined by the Brunauer–Emmett–Teller (BET) adsorption method (ASAP2460, Norcross, GA, USA). The type of iron supported by pomelo peel-based biochar and the surface functional groups of BC and MBC were analyzed by X-ray photoelectron spectroscopy (XPS). The magnetization of the samples was measured at room temperature using a vibrating sample magnetometer (VSM, Lake shore 7410, Westerville, OH, USA). The pH of biochar was determined as follows: biochar was mixed with ultrapure water at a mass ratio of 1:10, stirred magnetically for 0.5 h, and then placed for 1 h. Then, the pH of biochar was measured by a PHS-3C pH meter (thunder magnet, Shanghai, China). The zero point charge of biochar was measured by titration in accordance with reference [34].

### 2.4. Adsorption Experiments

A total of 0.01 g of BC, MBC, and 25 mL BPA solution were weighed in a 50 mL centrifuge tube, placed in a constant temperature oscillator in a water bath, and shaken in the dark at 150 rpm (to avoid possible BPA photodegradation). After a certain period of oscillation, the absorbance of the solution was measured at the wavelength of 276 nm by UV-visible spectrophotometer (UV-1810, Youke, Shanghai, China) after filtrating the 0.45 um membrane, and the concentration was converted into the established BPA standard curve [35]. In the single-factor experiment, the effects of the biochar dosage (0.1, 0.2, 0.3, 0.4, 0.5, and 0.6 g·L^−1^, BPA = 2 mg·L^−1^), adsorption time (10, 20, 30, 60, 90, 120, 150, and 180 min, BPA = 4 mg·L^−1^), initial BPA concentration (8, 12, 18, 26, 36, 50, 70, 100, 150, and 200 mg·L^−1^), reaction temperature (25, 35, and 45 °C), pH (3–10, BPA = 10 mg·L^−1^), Na^+^ concentration (0, 0.2, 0.4, 0.6, 0.8, and 0.10 mol·L^−1^, BPA = 20 mg·L^−1^) on the adsorption of bisphenol A by BC and MBC were investigated. In order to test the reusability of MBC, adsorption and desorption experiments were designed as follows: 0.0100 g of MBC was weighed in a 50 mL centrifuge tube. We added 25 mL, 100 mg·L^−1^ bisphenol A solution and shook it for 2 h. Then, MBC was separated with a magnet and the concentration of supernatant was determined. The separated biochar was soaked in an appropriate amount of ethanol, washed and dried overnight, and continued to the next adsorption test.

### 2.5. Data Analysis

The adsorption test was repeated three times for each group, and the mean value of the data was taken. Removal rate R (%) and adsorption capacity *Q_e_* (mg·g^−1^) were calculated by the following two formulas:

(1) $$R\left(\%\right)=\frac{c_{0}-c_{t}}{c_{0}}\times 100,$$

(2) $$\;Qe=\left(c_{0}-c_{t}\right)\times \frac{v}{m}\;,$$

where *C*_0_ (mg·L^−1^) is the initial concentration of bisphenol A. *C_t_* (mg·L^−1^) is the residual concentration of bisphenol A at time *t*. *V* (mL) is the solution volume. *m* (g) is the added mass of biochar.

The fitting model formulas adopted in this experiment are also listed as follows:

Pseudo-first-order kinetic equation:

(3) $$\ln\left(q_{e}-q_{t}\right)=lnq_{e}-k_{1}t;$$

pseudo-second-order kinetic equation:

(4) $$\frac{t}{q_{t}}=\frac{t}{q_{e}}+\frac{1}{k_{2}q_{e}^{2}};$$

Elovich equation:

(5) $$q_{t}=a+blnt;\;\mathrm{and}$$

intraparticle diffusion equation:

(6) $$q_{t}=k_{3}t^{\frac{1}{2}}+I,$$

where *q_e_* (mg·g^−1^) is the adsorption capacity of bisphenol A at the adsorption equilibrium. *Q_t_* (mg·g^−1^) is the adsorption capacity of bisphenol A at time *t*. *k*_1_ (min^−1^) and *k*_2_ [g·(mg^−1^·min)^−1^] are the adsorption rate constants of pseudo-first- and pseudo-second-order kinetic equations, respectively. A [mg·(g·min)^−1^] and *b* (g·mg^−1^) are the initial adsorption rate constants and desorption rate constants, respectively. *k*_3_ [mg·(mg·min^1/2^)^−1^] is the interparticle diffusion rate constant, which is related to the diffusion coefficient of particles. *I* is the constant related to the boundary layer thickness. Langmuir equation:

(7) $$Q_{e}=\frac{Q_{m}K_{L}C_{e}}{1+K_{L}C_{e}};$$

Freundlich equation:

(8) $$Q_{e}=K_{F}C_{e}^{\frac{1}{n}};$$

Temkim equation:

(9) $$Q_{e}=Aln\left(K_{T}C_{e}\right);\;\mathrm{and}$$

Dubinin–Radushkevich(D–R) equation:

(10) $$Q_{e}=Q_{0}\exp\left\{-0.5{\left[\frac{RTln\left(1+\frac{1}{C_{e}}\right)}{E}\right]}^{2}\right\},$$

where, *Q_e_* is the equilibrium adsorption capacity of bisphenol A (mg·L^−1^). *C_e_* is the solution concentration (mg·L^−1^) at adsorption equilibrium. *K_L_* is Langmuir isothermal adsorption constant (L·mg^−1^). *K_F_* (L·mg^−1^) and n are Freundlich equation constants. *Q_m_* is the theoretical maximum adsorption capacity (mg·g^−1^). A is the coefficient of the Temkim equation, which is related to the adsorption heat. *K_T_* is the equilibrium binding constant, mg·L^−1^. *Q*_0_ is the maximum unit adsorption, mg·g^−1^. *R* is the theoretical gas constant, 8.314 J·(mol·K)^−1^. *T* is the absolute temperature. *E* is the adsorption free energy, J·mol^−1^.

## 3. Results

### 3.1. Characterization of BC and MBC

Some physical and chemical properties of BC and MBC are shown in Table 2 [36]. Compared with BC, MBC showed that the composition of the C element decreased, while the contents of O and Fe increased. This indicates that in addition to loading Fe onto the surface of BC, more oxygen-containing functional groups are also introduced in the modification process. The specific surface area of MBC increased significantly from 1.706 m^2^·g^−1^ to 20.732 m^2^·g^−1^. This may be due to the high specific surface area of the loaded iron oxide nanoparticles, which significantly increases the surface area. The increase of the specific surface area is beneficial to the adsorption capacity of the adsorbent. At the same time, the pore volume and aperture of MBC are also larger than that of BC, which is more conducive to the adsorption of macromolecules.

**Figure 1:**
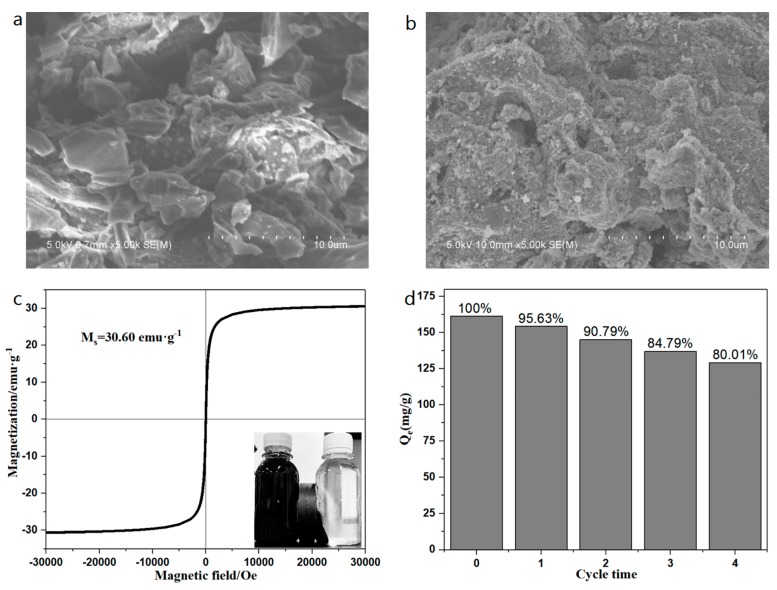
SEM image of BC (**a**) and MBC (**b**), magnetic hysteresis curve of MBC (**c**), reusability of MBC for BPA removal (**d**).

According to Figure 1a, b of the SEM of BC and MBC, it can be seen that the surface of BC is relatively smooth and the pore structure is not completely developed. However, the surface of MBC is relatively rough, with significantly more pores than BC, and the surface is loaded with many nano-iron oxide particles. To test the magnetic strength of the prepared magnetic biochar, the hysteresis loop of MBC was determined at room temperature (298.15 K). The results show that the hysteresis loop of MBC is symmetric about the origin and has obvious superparamagnetism [37]. Its saturation magnetization (Ms) is up to 30.60 emu·g^−1^. The lower right corner of Figure 1c shows the separation effect of BC and MBC after 1 min of an external magnetic field. It can be found that MBC has excellent magnetic separation ability. Figure 1d shows the adsorption of BPA by a MBC cycle for four times. After a cyclic adsorption for four times, the adsorption capacity of bisphenol A by MBC still reached about 80% of the initial adsorption capacity, indicating that the experimental MBC had a good cyclic adsorption performance. The reason for the adsorption capacity decrease may be that during multiple adsorption–desorption processes, some of the nano-iron oxide particles and active substances on the surface of MBC were eluted, resulting in a decrease of the adsorption capacity. Table 3 shows the adsorption capacity of bisphenol A and the magnetic strength of the magnetic materials prepared in previous literatures.

**Figure 2:**
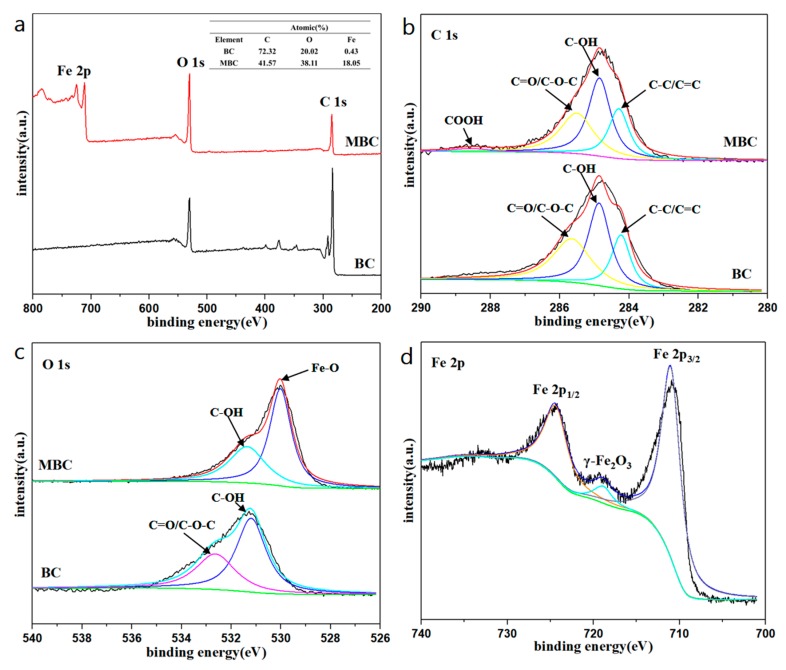
(**a**) XPS spectra of BC and MBC, and XPS patterns of the (**b**) C 1s, (**c**) O 1s, (**d**) Fe 2p.

Figure 2 shows the XPS analysis spectrum of BC and MBC. It can be found from the XPS spectrum (a) that the biochar before and after modification has a similar XPS peak shape, and the peak at the binding energy of 284.8 eV corresponds to the C element in BC and MBC. After modification, the strength peak of the C element in MBC is weakened, while that of the O element is enhanced. The C 1s spectrum showed that both the surface of BC and MBC contained a large number of oxygen-containing functional groups, including C-C/C=C, C-OH, C=O/C-O-C, COOH, etc. Compared with BC, the peak area of C-C/C=C decreases, the peak area of C-OH and C=O/C-O-C increases, and COOH appears. This indicates that in the process of magnetic modification, more oxygen-containing functional groups are introduced on the surface of BC. The O 1s spectrum showed that the C element in BC mainly existed in the form of organic carbon (531.75 and 533.02 eV) [43]. The peak area of C-OH and C=O/C-O-C of MBC decreased compared with that of BC, while the peak area of Fe-O increased significantly, and the peak of O element shifted from 531.80 eV to 530.03 eV, closer to the Fe-O peak. This indicates that the introduced Fe is not loaded on the surface of BC in the form of hydroxide. In order to determine the phase of iron oxide, the Fe 2p spectrum of MBC is shown in Figure 2d. The two main peaks at 710.7 eV and 724.3 eV correspond to Fe 2p_3/2_ and Fe 2p_1/2_, respectively. The satellite peak at 719.3 eV is the characteristic peak of γ-Fe_2_O_3_ [44]. Moreover, the surface of MBC is slightly red. It indicates that the iron oxide supported on the pomelo peel-based biochar is γ-Fe_2_O_3_.

### 3.2. Effects of Biochar Dosage

**Figure 3:**
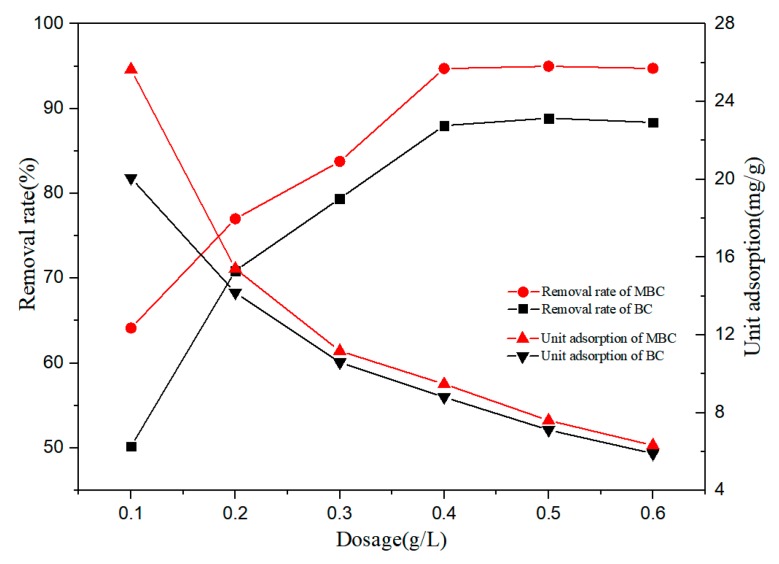
Effects of BC and MBC dosage on BPA adsorption.

The effect of biochar dosage on the adsorption of bisphenol A by BC and MBC is shown in Figure 3. The removal rate of bisphenol A increased with the increase of the biochar dosage. When the biochar dosage was 0.1 g·L^−1^, the removal rates of bisphenol A by BC and MBC were 50.19% and 64.13%, respectively. When the biochar dosage increased to 0.4 g·L^−1^, the removal rate increased significantly, 88.01% and 94.74%, respectively. Subsequently, the biochar dosage was increased, and the removal rate remained unchanged. The increased removal rate can be attributed to the increased biochar, which not only increases the effective specific surface area of adsorption, but also increases the active site of adsorption [45]. At the same time, the unit adsorption of bisphenol A by BC and MBC decreased gradually. This is because in the case of limited solutes, too many adsorbents will not only compete for solutes, but also overlap the effective adsorption active sites on the biochar surface [46].

### 3.3. Effects of Time

**Figure 4:**
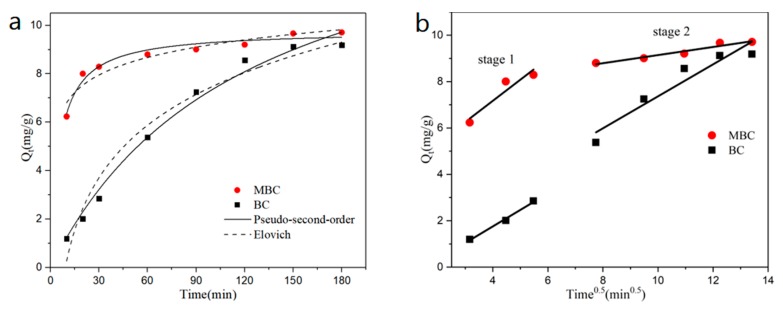
Effects of the adsorption equilibrium time on BPA adsorption and kinetic equation fitting: pseudo-second-order (P-S-O) and Elovich (**a**); intraparticle diffusion (**b**).

The effect of adsorption time on the adsorption of bisphenol A by BC and MBC is shown in Figure 4a. The adsorption of bisphenol A by the two biochars reached equilibrium at around 150 min. Compared with BC, MBC can reach more than 87% (8.5420 mg·g^−1^) of the saturated adsorption capacity (9.7098 mg·g^−1^) within 30 min of the initial adsorption stage, which is significantly faster than BC. In order to further explore the adsorption process of bisphenol A by BC and MBC, pseudo-first-order and pseudo-second-order kinetic models, Elovich model, and the intraparticle diffusion model are used to fit the experimental data. The fitting curve is shown in Figure 4, and the parameter fitting results are shown in Table 4.

From the fitting results, it can be seen that the pseudo-second-order kinetic model has the best fitting effect on the adsorption of bisphenol A by BC and MBC, and the correlation coefficient is above 0.98, indicating that chemical adsorption is the rate control step of the adsorption process, and the adsorption mechanism is controlled by a variety of forces (such as π–π electron donor–acceptor interaction, H-bond, etc.). The Elovich model is commonly used to describe the adsorption behavior of adsorbents on non-uniform solid surfaces, while revealing data irregularity that is ignored by other dynamic models [47]. The correlation coefficient of BC and MBC fitted by the Elovich model is above 0.90, indicating that the adsorption of BC and MBC to bisphenol A is a chemical adsorption process of a non-uniform solid adsorbent, with evenly distributed surface adsorption energy [48]. The analytical constant b of bisphenol A adsorption by MBC is smaller than that of BC, indicating that the adsorption of bisphenol A by MBC is more stable [49]. In this experiment, the intraparticle diffusion model of bisphenol A by BC and MBC was fitted into two stages. The first stage is the diffusion of BPA through the liquid membrane to the surface of the adsorbent. The second stage is the adsorption of BPA on the surface of the adsorbent. The fitting effect of the first stage of MBC was poor (0.8045), the fitting effect of the second stage was good (0.9289), and the effect of BC was opposite. This indicated that magnetic modification affected the adsorption of bisphenol A by MBC, accelerated the adsorption rate of bisphenol A, and reduced the adsorption resistance of bisphenol A [50]. The fitting parameters of the intraparticle diffusion model of BC and MBC are *k_d_*_1_ < *k_d_*_2_ and I_1_ < I_2_, indicating that the adsorption rate gradually decreases while the adsorption resistance gradually increases during the adsorption process [51]. At the same time, it can be seen from Figure 4b that the fitted curves have not passed through the origin, indicating that the diffusion in particles is not the only rate control step, and the adsorption rate is also affected by chemical forces, such as ion exchange and H-bond [52].

### 3.4. Effects of Initial BPA Concentration and Temperature

**Figure 5:**
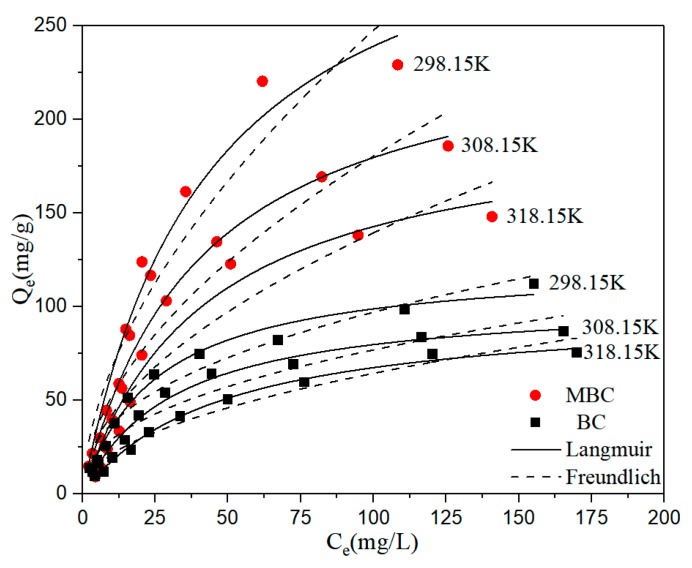
Adsorption isotherm fitting of BPA by BC and MBC.

The effect of initial BPA concentration on the adsorption of bisphenol A by BC and MBC is shown in Figure 5. With the increase of initial BPA concentration, the adsorption capacity of BC and MBC to bisphenol A also increased gradually. This is because the increased concentration of bisphenol A can enhance the adsorption drive between the adsorbent and the solute [53,54]. Four common adsorption isothermal models are used to further study the adsorption behavior and mechanism. The fitting results are shown in Figure 5, and the relevant fitting parameters are shown in Table 5; Table 6.

The Langmuir model best describes the adsorption isotherm of bisphenol A adsorbed by BC and MBC, and the correlation coefficient is above 0.95. The Freundlich fit is also good. This indicates that bisphenol A has not only monolayer adsorption on BC and MBC, but also multi-molecular adsorption. It is speculated that the reason may be that the load of γ-Fe_2_O_3_ on the surface of BC causes the surface of MBC to be rougher; thus, multi-molecular layer adsorption occurs locally. In addition, the 1/n of the Freundlich model represented the uniformity of the material surface, while the 1/n value of MBC fitting was greater than BC, confirming this view. The A value in the Temkim equation represents the adsorbent coverage, while the A value of the BC adsorbed BPA fitting is smaller than that of the MBC fitting, indicating that the theoretical adsorbent coverage of BPA on BC is higher than that of MBC, that is, the electrostatic attraction between BC and BPA is stronger than MBC [55]. The parameter E of the D–R model is the characteristic energy. When E < 16 kJ·mol^−1^, the adsorption was mainly physical adsorption. When E > 40 kJ·mol^−1^, the adsorption was mainly chemical adsorption. The fitted E values were >40 kJ·mol^−1^, indicating that the adsorption process was dominated by chemical adsorption. By calculating the equilibrium parameter R*_L_*, we can judge whether the adsorption process is favorable [56]. (0 < R*_L_* < 1 is a favorable adsorption. R*_L_* > 1 is an unfavorable adsorption. R*_L_* = 1 is a linear adsorption. R*_L_* = 0 is a irreversible adsorption.) The calculation formula is as follows, and the meanings of parameters are the same as above:

(11) $$R_{L}=\frac{1}{1+K_{L}C_{0}}.$$

The calculation shows that the R*_L_* values of BC and MBC are within the range of 0~1. Therefore, the adsorption of bisphenol A by BC and MBC is favorable.

The effect of temperature on the adsorption of bisphenol A by BC and MBC is shown in Figure 5. With the increase of temperature, the adsorption capacity of bisphenol A by BC and MBC decreased. In order to further explore the thermodynamics of adsorption of bisphenol A by BC and MBC, Equations (12) and (13) are used to calculate the adsorption data at different temperatures to calculate the gibbs free energy (ΔG), enthalpy (ΔH), and entropy (ΔS) in the adsorption process. The calculation formula is as follows:

(12) $$\Delta G=-{\mathrm{RTlnK}}_{e},$$

(13)  ΔG=ΔH−TΔS ,

where R is the gas constant, 8.314 J·(mol·K)^−1^. T is temperature, K. lnK*_e_* is the equilibrium constant. The results are listed in Table 7. The ΔH values are negative, indicating that the BC and MBC adsorption process of bisphenol A is exothermic. It is generally believed that when ΔH values are within the range of 2.1–20.9 kJ·mol^−1^, the adsorption process is mainly dominated by physical adsorption [57]. While ΔH values are not in the range of this study, the BC and MBC adsorption process of bisphenol A is mainly dominated by chemical adsorption. ΔG is less than 0 and decreases with the decrease of temperature, indicating that the BC and MBC adsorption reaction of bisphenol A is a spontaneous exothermic reaction. ΔS < 0 reveals a decrease in the disorder between the adsorbent and the solute during the adsorption of BPA by BC and MBC [58].

### 3.5. Effects of Solution pH and Ionic Strength

**Figure 6:**
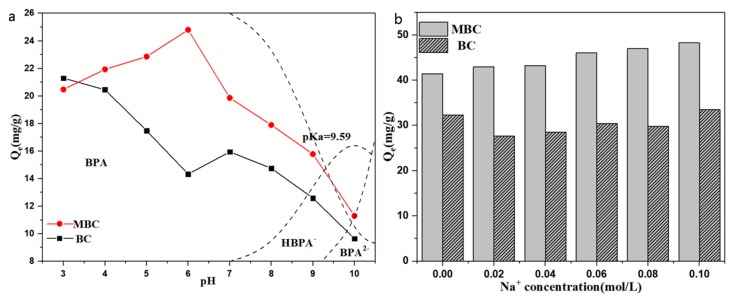
Effects of pH (**a**) and Na^+^ concentration (**b**) on the adsorption of BPA by BC and MBC.

Figure 6a shows the effect of solution pH on the adsorption of bisphenol A by BC and MBC. As can be seen from the figure, the adsorption of bisphenol A by BC and MBC is strongly pH dependent. The maximum adsorption capacity of BC to bisphenol A occurs at pH = 3, which is due to the formation of π–π electron donor–acceptor interaction (EDA) between BC and bisphenol A, accompanied by a strong H-bond [59,60]. When pH increased from 6 to 7, the adsorption capacity of BC to bisphenol A rose again. This is because bisphenol A begins to partially dissociate, and bisphenol A in the solution is no longer in molecular form, but part of HBPA^−^ appears. The surface of BC is positively charged, and the electrostatic attraction between BC and HBPA^−^ enhances the adsorption. The subsequent increase in pH resulted in a further decrease in the adsorption capacity of BC to bisphenol A. This is because the π–π electron donor–acceptor interaction (EDA) and hydrogen bonding between BC and BPA is weakened when the pH of the solution is greater than the acid dissociation constant of BPA [61]. The adsorption capacity of bisphenol A by MBC increased at first and then decreased with the increase of pH, and the maximum adsorption capacity occurred at pH = 6. The zero point charge of MBC, pH_pzc_, is 5.86. When the pH is less than 6, the dissociation of BPA increases with the increase of pH. The increase in HBPA^−^ leads to increased electrostatic attraction between BPA and positively charged MBC surfaces. In addition, protonated phenol -OH on BPA under acidic conditions will also generate electrostatic repulsion with MBC, which is not conducive to the adsorption of BPA by MBC. When the pH continues to rise, BPA further dissociates to produce the more electronegative BPA^2−^, which generates a strong electrostatic repulsion with the negatively charged MBC surface, resulting in a decrease in the adsorption capacity.

As shown in Figure 6b, with the increase of NaCl concentration, the adsorption capacity of BC to BPA decreased at first and then increased. When the Na^+^ concentration was 0.10 mol·L^−1^, the adsorption capacity was almost the same as when NaCl was not added. However, the increase of Na^+^ concentration significantly enhanced the adsorption capacity of BPA to MBC. The addition of NaCl not only makes BPA exist in more molecular states (salting out effect), but also deeply penetrates into the diffusion layer on the adsorbent surface, weakens the repulsive effect between adsorbents, and makes the adsorbents more compact (squeezing out effect). Among them, the “salting out effect” will enhance the hydrophobic effect between BC, MBC, and BPA to enhance the adsorption effect, while the “squeezing out effect” will reduce the effective adsorption area of the adsorbent and weaken the adsorption of BC and MBC to BPA. Therefore, the Na^+^ concentration is in the range of 0–0.10 mol·L^−1^, and the two effects combined show that the adsorption capacity of BC adsorbed BPA is inhibited first and then recovered. However, the specific surface area of MBC (20.732 m^2^·g^−1^) is much higher than that of BC (1.706 m^2^·g^−1^), and the “squeezing out effect” has little influence on MBC, so it is shown as a promotion effect.

### 3.6. Mechanism of the Adsorption of BPA by BC and MBC

**Figure 7:**
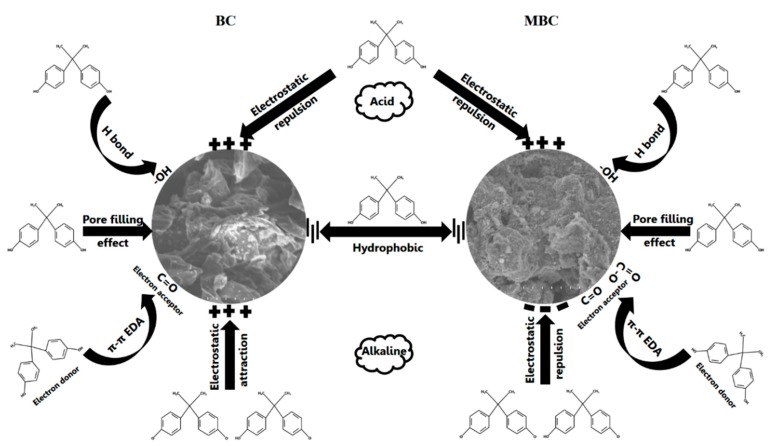
Mechanism of adsorption of BPA by BC and MBC.

Figure 7 is a schematic diagram of the mechanism of the adsorption of BPA by BC and MBC. The average pore size of BC and MBC is between 2 and 50 nm, which is mesoporous. BPA molecules can enter the pores of BC and MBC through pore filling. As mentioned above, the adsorption capacity of BC and MBC to BPA is greatly affected by pH. Under acidic conditions, the phenolic hydroxyl groups in the structure of BPA protonize, generating electrostatic repulsion with the positively charged surfaces of BC and MBC. The functional group -OH on the surface of BC and MBC also forms strong hydrogen bonds with the -OH and C-H on the molecular structure of BPA. In addition, acidic functional groups of BC and MBC (such as -COOH, C=O, etc.) can act as electron acceptors, forming π–π electron donor–acceptor interactions (EDA) with BPA.

Under alkaline conditions, the surface of MBC is converted into a negative charge, which maintains electrostatic repulsion with the dissociated HBPA^−^ and BPA^2−^, while BC is the opposite. In addition, the hydrogen bonds between BC, MBC, and BPA, as well as the π–π electron donor–acceptor interaction (EDA), would be greatly weakened, making it difficult for BC and MBC to absorb bisphenol A. Bisphenol A has strong hydrophobicity and can be combined with the hydrophobic site on the surface of BC and MBC. Therefore, a hydrophobic interaction is also an important driving force for BC and MBC to absorb BPA.

## 4. Conclusions

In this paper, grapefruit peel waste was used as a biochar source to prepare biochar and magnetize it. Through characterization, the modified magnetic biochar was found to have a larger specific surface area, larger pore volume, and more oxygen-containing functional groups. The prepared magnetic biochar is loaded with nano γ-Fe_2_O_3_ particles, and thus possessing excellent magnetic separation ability. BC and MBC were used to adsorb BPA in simulated wastewater, and it was found that the adsorption kinetics and adsorption isotherm were in accordance with the pseudo-second-order kinetics model and Langmuir model, respectively. The adsorption of BPA by BC and MBC is a spontaneous exothermic reaction with strong pH and ionic strength dependence. The study of the adsorption mechanism shows that the H-bond and the π–π electron donor–acceptor interaction (EDA) are the main forces for BC and MBC to adsorb BPA. After cyclic adsorption of BPA for four times, the adsorption capacity of MBC to BPA can still reach about 80% of the initial adsorption capacity, with good recycling and reusability.

## Figures and Tables

**Table 1:** Characteristics of bisphenol A (BPA).

Molecular Formula	Molecular Mass	pK_a_	Lipid–Water Partition Coefficient (lgP)	Chemical Structure
C_15_H_16_O_2_	228.29	pK_a_ = 9.59	3.32	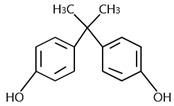

**Table 2:** Physico-chemical characteristics of biochar (BC) and magnetic biochar (MBC).

	C(%)	O(%)	Fe(%)	pH	pH_pzc_	Specific Surface Area (m^2^/g)	Pore Volume (cm^3^/g)	Pore Diameter (nm)
**BC**	72.32	20.02	0.43	10.13	10.98	1.706	0.003	5.896
**MBC**	41.57	38.11	18.05	7.96	5.86	20.732	0.110	20.897

**Table 3:** Comparison of BPA removal by reported adsorbents in the literature and this study.

Adsorbent	Experimental Conditions	Q_max_ (mg/g)	M_s_ (emu/g)	Reference
**Molecularly imprinted polymers based on magnetic graphene oxide**	500 mg·L^−1^ BPA 100 mg·L^−1^ adsorbent	106.38	6.12	Wang et al. [38]
**Polyethersulfone magnetic microspheres**	68 mg·L^−1^ BPA 300 mg·L^−1^ adsorbent	63.24	17.78	Yu et al. [39]
**Magnetic microbeads**	300 mg·L^−1^ BPA 100 mg·L^−1^ adsorbent	139.6	Not mentioned	Osman et al. [40]
**Magnetic CuZnFe_2_O_4_-biochar**	80 mg·L^−1^ BPA 200 mg·L^−1^ adsorbent	263.2	37.6	Heo et al. [41]
**Magnetic composite organic sepiolite**	50 mg·L^−1^ BPA 1000 mg·L^−1^ adsorbent	36.30	14.1	Yang et al. [42]
**Magnetic grapefruit peel biochar**	200 mg·L^−1^ BPA 400 mg·L^−1^ adsorbent	229.19	30.60	Our study

**Table 4:** Fitting parameters of four kinetic models for adsorption of BPA onto BC and MBC.

Kinetic Model	Parameters	BC	MBC
**Pseudo-first-**	*k*_1_/min^−1^	0.0114	0.0917
**order kinetic**	*Q_e_*/mg·g^−1^	10.9917	9.5010
	R^2^	0.9922	0.9066
**Pseudo-second-**	*k*_2_/g·(mg·min)^−1^	0.0005	0.0155
**order kinetic**	*Q_e_*/mg·g^−1^	16.2909	10.1574
	R^2^	0.9870	0.9877
**Elovich**	*a*/mg·(g·min)^−1^	−6.9359	4.0059
	*b*/g·mg^−1^	3.1293	1.1806
	R^2^	0.9625	0.9065
**Intraparticle diffusion**			
	*k_d_*_1_/mg·(mg·min^1/2^)^−1^	0.7115	0.9109
**Stage 1**	I_1_	−1.0899	3.5282
	R^2^	0.9857	0.8045
	*k_d_*_2_/mg·(mg·min^1/2^)^−1^	0.6894	0.1749
**Stage 2**	I_2_	0.4711	7.3938
	R^2^	0.8883	0.9289

**Table 5:** Fitting parameters of isotherm fitting for BPA adsorption onto BC.

Isotherm Models	Parameters	298.15 K	308.15 K	318.15 K
**Langmuir**	*K_L_* (L·mg^−1^)	0.0394	0.0324	0.0213
	*Q_m_* (mg·g^−1^)	123.8331	104.2741	99.0099
	R^2^	0.9840	0.9830	0.9938
**Freundlich**	*K_F_* (L·mg^−1^)	14.2885	10.8794	6.8863
	1/n	0.4159	0.4244	0.4851
	R^2^	0.9542	0.9286	0.9596
**Temkim**	A	24.7169	21.1330	19.7639
	*K_T_* (mg/L)	0.4937	0.3899	0.2697
	R^2^	0.9755	0.9626	0.9713
**D–R**	*Q_m_*/mg·g^−1^	92.0233	78.4427	67.6033
	E/kJ·mol^−1^	159.5204	121.9018	100.8157
	R^2^	0.8496	0.8812	0.8318

**Table 6:** Fitting parameters of isotherm fitting for BPA adsorption onto MBC.

Isotherm Models	Parameters	298.15 K	308.15 K	318.15K
**Langmuir**	*K_L_* (L·mg^−1^)	0.0232	0.0246	0.0238
	*Q_m_* (mg·g^−1^)	342.4692	252.5538	202.7956
	R^2^	0.9724	0.9552	0.9416
**Freundlich**	*K_F_* (L·mg^−1^)	18.1606	14.9137	12.7045
	1/n	0.5674	0.5413	0.5201
	R^2^	0.9155	0.8924	0.8576
**Temkim**	A	62.7503	55.4817	45.5962
	*K_T_* (mg/L)	0.3427	0.2484	0.2246
	R^2^	0.9219	0.9730	0.9555
**D–R**	*Q_m_*/mg·g^−1^	219.6894	169.1809	141.9508
	E/kJ·mol^−1^	119.1388	131.5717	114.5335
	R^2^	0.9325	0.9540	0.9667

**Table 7:** Thermodynamic parameters of BPA adsorption onto BC and MBC at various temperatures.

	T/K	lnK_e_	∆G/kJ·mol^−1^	∆H/kJ·mol^−1^	∆S/J (mol·K)^−1^
	298.15	1.3541	−3.3566		
**BC**	308.15	0.9884	−2.5322	−29.6316	−88.0668
	318.15	0.6021	−1.5926		
	298.15	1.8341	−4.5464		
**Fe_2_O_3_@BC**	308.15	1.3939	−3.5711	−28.6981	−81.1800
	318.15	1.1078	−2.9302		
